# The evolution, progress, and future direction of Nepal's universal salt iodization program

**DOI:** 10.1111/mcn.12945

**Published:** 2020-02-03

**Authors:** Naveen Paudyal, Stanley Chitekwe, Sanjay Rijal, Kedar Parajuli, Chandrakant Pandav, Macha Maharjan, Robin Houston, Jonathan Gorstein

**Affiliations:** ^1^ Nutrition Department UNICEF Nepal Kathmandu Nepal; ^2^ Family Welfare Division, Ministry of Health and Population Government of Nepal Kathmandu Nepal; ^3^ Iodine Global Network New Delhi India; ^4^ Iodine Global Network Kathmandu Nepal; ^5^ Iodine Global Network Ottawa Ontarion Canada

**Keywords:** Iodine deficiency, micronutrients, Nepal, salt iodization

## Abstract

Nepal is located in what was once known as the Himalayan Goitre Belt and once had one of the highest prevalence's of iodine deficiency disorders in the world. However, through a well‐executed universal salt iodization program implemented over the past 25 years, it has achieved optimal iodine intake for its population, effectively eliminating the adverse consequences of iodine deficiency disorders. A comprehensive review of policy and legislation, surveys, and program reports was undertaken to examine the key elements contributing to the success of this program. The paper reviews the origins and maturation of salt iodization in Nepal, as well as trends in the coverage of iodized salt, the iodine content in salt, and population iodine status over the past two decades. The paper describes critical components of the program including advocacy efforts, trade issues with India, the role of the Salt Trading Corporation, monitoring, and periodic program reviews. The paper discusses the recent findings from the 2016 national micronutrient survey demonstrating the success of the salt iodization program and describes emerging challenges facing the program in the future.

**Key points**

- Nepal has achieved adequate iodine intake through a successful salt iodization program.
- The recent micronutrient survey confirms this success, demonstrating high use of adequately iodized salt and adequate iodine intake all population groups sampled.
- The program success is based on ongoing advocacy, strong legislation mandating iodization, routine monitoring at border stations, consumer education through communication campaigns, and periodic program reviews.
- Future challenges include managing the iodine content in iodized salt to salt to address excessive iodine in salt, understanding use of iodized salt in processed foods, and insuring program visibility to ensure sustainability.

## 1 INTRODUCTION

In 2016, Nepal conducted a nationally representative micronutrient status survey, which provided evidence of the remarkable progress that the country has made towards the reduction of vitamin A deficiency, anaemia, and improvement in iodine status (UNICEF, 2016). The survey provided updated estimates on the coverage and reach of large‐scale interventions, including universal salt iodization (USI) as reflected by the proportion of households with access to adequately iodized salt (HHIS). These data indicate that Nepal has achieved optimal iodine intake through USI, thus reducing the risk of cognitive impairment among newborns born today and enabling children to thrive. This success is the result of a number of critical factors, including early recognition of the problem, political commitment, establishment of an exclusive agency responsible for the importation, storage and distribution of iodized salt from India (the Salt Trading Corporation [STC]), development of strategic plans of action that defined priority actions, early adoption of legislation that stipulated that all edible salt be iodized (USI), periodic surveys that tracked performance and implementation, and the deployment of targeted communications to encourage consumer demand to shift towards a higher quality product with adequate iodine levels. Furthermore, these elements are well established within the national nutrition architecture, which will contribute towards the sustainability of this success, including mandatory salt iodization, monitoring of imported salt, inclusion of iodine in the multisectoral nutrition strategy, and continuing to track iodine intake, as was done in the recent survey.

As with any success, future changes and revisions need to be anticipated. Consumer preferences can change. Salt reduction strategies may lead to an overall decrease in the intake of salt, whereas dietary patterns lead to an increased proportion of salt consumed in processed foods and condiments instead of on a discretionary basis. Although there is currently no monitoring system tracking salt consumption patterns, this will become increasingly important to determine whether to adjust the iodine standards in iodized salt. Political will may shift away from nutrition, and anti‐regulatory forces may change the ability to ensure the exclusive import of iodized salt. Weakened monitoring systems may allow noniodized or inadequately iodized salt to enter Nepali markets. The purpose of this paper is to highlight the remarkable success Nepal has achieved and the key factors responsible, while discussing future directions for ensuring and sustaining optimum iodine nutrition in Nepal.

## 2 METHODS

The paper is based on a comprehensive review of past and current surveys, research papers, legislation, trade documents, policy statements, and national strategic plans. The review focused on key program elements, including political commitment, partnership, monitoring systems, social mobilization, and trade issues. Trend analysis for key indicators is based on four nationally representative surveys conducted in 1998 (Government of Nepal, 1998), 2005 (Government of Nepal, 2005), 2007 (Government of Nepal, 2007), and 2016 (Government of Nepal, 2016), which characterized and captured critical changes in USI program performance resulting from programmatic adjustments and refinements. In addition, a number of subnational surveys have also been reviewed and discussed. For review of salt iodine content, only reports and papers that have utilized titration method have been selected, and findings based on rapid test kit method have been excluded.

The findings will focus on historical context, salt iodization program implementation, monitoring salt iodization consistency, marketing to improve uptake, and trends in key indicators and impact on status.

## 3 RESULTS

### 3.1 The early years: Recognizing the problem of IDD and adopting the challenge of USI

Iodine deficiency was recognized very early in South Asia, including Nepal. Perhaps as early as 200 BC, goitre was described in Indian medical texts, and Chinese sources suggest use of marine plants for its treatment as early as 100 BC. Reports from British visitors to Nepal in the late 1700's noted a high prevalence of goitre and cretinism—attributed to a wide variety of causes by the local population. Work in India began to focus on iodine as a treatment, and in 1832, Joseph Bramley, the Medical In‐Charge at the British Residency in Kathmandu, presented on use of iodine in the treatment of goitre. There were accounts of tens of thousands of cases of goitre treated in the Nepal Terai by British medical officers in the ensuing years (Miles, 1998).

The world began to recognize the importance of iodine deficiency and its global distribution in the mid 1980's, as research studies established links between suboptimal iodine and brain development, although a large number of surveys demonstrated significant iodine deficiency in many more countries than expected. (Delange, 2000; Hetzel & Pandav, 1996). With the World Summit for Children in 1990, iodine deficiency was established as a critical public health issue. This was followed by a declaration by the 43rd World Health Assembly, which stated that “WHO shall aim at eliminating iodine deficiency disorders as a major public health problem in all countries by the year 2000” (World Health Organization, 1990).

Nepal responded quickly to the challenge of elimination in response to early subnational surveys showing high goitre rates. Although Nepal imported iodized salt from India as early as 1973 and initiated iodized oil supplementation in target districts in 1979, small surveys continued to show severe iodine deficiency, including one in 1980 that “found 107 cretins and/or deaf mutes in a population of 2,456 persons in the northernmost community (of Dolakha District) bordering Tibet” (Achard, 1987). The magnitude of the problem was even reported in the New York Times, commenting on the “Himalayan goiter belt,” mentioning the injection program, and the early but ineffective salt iodization in India (Eckholm, 1985). Clearly, there was strong interest in the problem of goitre and cretinism both in Nepal and India, but early on, the challenges of addressing the problem at the national level seemed overwhelming. Experts suggested that “the undoubted progress that has occurred in understanding goitre and cretinism, and knowledge of their treatment has yet to be universally applied. To do so is technically feasible in South Asia, though questions remain on epidemiology, and there are serious doubts whether the sociopolitical will exists to tackle the problem” (Miles, 1998).

### 3.2 Establishing national programs: Addressing goitre and establishing importation of iodized salt

By the early 1970's, the Government of Nepal recognized that iodine deficiency disorder (IDD) was a serious public health problem. In 1973, the government established the first national IDD control program, which consisted of two components/project elements: The Goitre Control Project (GCP) and the Goitre and Cretinism Eradication Project (GCEP) under the Ministry of Health (the salt iodization program under GCP were transferred to the Ministry of Commerce and Supplies in 1998). The primary objective of the GCEP was to provide “intensive efforts” to address IDD through iodized oil supplementation, whereas the GCP was designed to “coordinate and manage” all activities related to the implementation and monitoring of salt iodization. (Government of Nepal, 2013)

The primary intervention of the GCEP was initially the provision of iodized oil injections and capsules. From 1979–1994, the program supported iodized oil injections in 40 mountain and hill districts, shifting to the distribution of iodized oil capsules in 15 remote districts between 1995 and 1998. (Poudel, 2016). The iodized oil supplementation program in Nepal was successful in reducing the prevalence of goitre and cretinism and increasing urinary iodine levels in mountain and hill districts, where access to iodized salt was negligible. The cost of iodine supplementation was much more expensive than salt iodization, and the lack of adequate public health infrastructure required for its delivery limited the reach of the intervention. As access to iodized salt increased considerably in remote parts of the country, iodine supplementation was phased out in 1998, adopting salt iodization as the sole strategy to control and eliminate IDD in the country. At the same time that supplements were being provided, there was increased attention to the viability of importing iodized salt from India to increase the supply and availability of iodine for the Nepali population. Because there is no salt produced in Nepal, the USI program leveraged the bilateral trade relationship with the government of India.

Early relations between India and Nepal were guided by the Treaty of Sagauli, established in 1816 during British rule in India. In 1950, this treaty was changed, and relations functioned under two treaties, one of which (the Treaty of Trade and Commerce) “recognized Nepal's right to import and export commodities through Indian territory and ports.” Given Nepal's geography, this treaty was critical for the supply of goods to the country. Diplomatic issues over the years have resulted in restrictions of this flow of goods from India, such as the closure of entry points by India in 1989, although this did not hinder salt availability due to a policy to have a 6‐month buffer stock. (Nations Encyclopedia, 1991)

Virtually, all salt consumed in Nepal comes from India, with some entering through China for some areas where road access to China is close. As early as 1963, the STC was established as a public–private partnership to manage import and distribution of salt. Although this early effort did establish a mechanism to import and distribute salt, little attention was given to the quality of iodization, either at the point of production in India or upon importation to Nepal.

The global attention on IDD and its elimination through USI intensified in the mid‐1980s and recognizing the cost and logistical difficulties with the injection program, Nepal began to focus increasingly on ensuring that all salt imported from India was iodized. Salt iodization units were established at five key border entry points, which iodized inadequately iodized shipments. In addition, in the 1980s, the STC started packaging and marketing refined and crushed iodized salt in 1‐kg consumer packages. The salt trade in Nepal has been regulated by several legislative acts. On the basis of the Export and Import (Control) Act, 2013 (1957) and the Competition Promotion and Market Protection Act, 2063 (2007), the Nepal government has authorized STC Ltd as the sole agency to import and distribute iodized salt throughout the country (Salt Trading Corporation, 2018). Salt quality standards were determined by the Food Act of 1967. In 1998, Nepal passed the Iodized Salt (Production, Sale and Distribution) Act, 2055(1998), which outlined the control, licencing, standards and oversight for importation of iodized salt, and in essence made iodization of all salt intended for human consumption mandatory. There is limited importation of noniodized salt for nonfood industrial applications, but the STC maintains strict control over the salt imported for edible purposes. This act established the regulatory mechanisms for USI and engaged partners in the development of communication efforts to educate the population about the importance of iodine deficiency and its prevention through the use of iodized salt. (Government of Nepal, 1998) It also established a high‐level government committee to oversee the elimination of IDD as a public health problem. Although passed, this act was to commence upon notification in the Nepal Gazette. No notification has been published, and regulations for the act are drafted but also not yet in the Gazette, leaving the act not legally operationalized.

Most of the salt in Nepal arrives by rail from a selection of producers in India, which are regulated by the Salt Commissioner's Office in India. Salt is a priority transport good in India, and some tax exemptions apply. (Clear Tax, 2018) This impacts the situation in Nepal by reducing the cost. Furthermore, the India rail system regulated the movement of salt, making compliance with iodization standards more likely and, thus, more likely for Nepal imports.

In 1997, Nepal established its first “Five‐year National Plan of Action for the Elimination of Iodine Deficiency Disorders” (1997–2002). This plan emphasized six priority actions, including strengthening legislation, establishing a national awareness campaign, establishing internal quality assurance with external verification carried out by the Department of Food Technology And Quality Control, the government agency responsible for implementing food regulations in Nepal, proposing periodic survey assessment, and increasing availability of adequately iodized salt throughout the country. The plan facilitated the development of an additional six 20,000 MT warehouses within the STC for the storage of salt in partnership with the Japan International Cooperation Agency, as well as many distribution depots/dealers throughout the country. The plan also initiated the “Iodized Salt Social Mobilization Campaign” with support from United Nations Children's Fund (UNICEF) and included a distribution program that included a continuation of transport subsidy for salt to 22 remote districts.

In 1998, the Nepal National Micronutrient Status Survey demonstrated that the USI program had indeed led to a marked improvement in both household iodized salt use and iodine status, and the findings triggered discontinuation of the iodized capsule program and renewed focus on further improving the USI program. Thus, by 1999, the national IDD plan recognized salt iodization as the sole intervention for addressing IDD elimination goals.

In 2002, the global community reaffirmed global IDD goals during the UN General Assembly Special Session on Children, moving the target for sustained elimination of IDD to 2005. (United Nations, 2002) Many countries, including Nepal, had made substantial progress in expanding universal salt iodization and increasing household use of iodized salt. The legislative and regulatory environment continued to improve. In 2001, the Nepal food standard established the standard for iodized salt: minimum 50 ppm at the production level and minimum 30 ppm at the retail level (Nepal Gazette, 2001). Although there remains a standard for noniodized salt, STC is authorized by the government as the sole agency to import and distribute only iodized salt (under the Export and Import [Control] Act and the Competition Promotion and Market Protection Act).

The next “Five Year National Plan of Action to Achieve Optimal Iodine Nutrition in Nepal” (2013–2017) focused on a series of program bottlenecks. This plan was designed to address five specific challenge areas, including strengthening the national IDD coordination structure, improving iodized salt coverage in hard to reach areas, increasing awareness of the importance of refined packaged salt, reducing losses in large crystal salt, and understanding changing patterns of consumption and use.

### 3.3 Improving the consistency of iodization

Longstanding cultural preference in Nepal was for coarse, unrefined salt called “Phoda” salt—in 1998, less than 10% of the population used refined salt and nearly 65% of the Nepali population (80% of the hill population) used phoda salt with the balance consuming crushed salt. (Government of Nepal, Ministry of Health, 1998). Phoda salt is packed in 50‐kg bags and usually sold in loose form in markets. Although this salt was iodized, the physical characteristics, transport, packaging, and storage practices all risked loss of iodine, thus contributing to inconsistent iodine content and thus inconsistent iodine intake.

As part of the national USI program, the government established a system for monitoring of the iodine content of salt. This consisted of monitoring by STC of salt in the warehouses storing salt imported from India. In addition, the Ministry of Health and Department of Food Technologies and Quality Control/Ministry of Agriculture and Livestock Development undertake regulatory monitoring of iodized salt that is focused mainly on retail level. To supplement these data, Nepal assessed the coverage of household iodized salt through periodic national surveys including DHS, Living Standard Surveys, MIC surveys, and specific micronutrient surveys. These efforts provided a strong database through which to review trends overtime and to identify gaps and opportunities.

Initial communications efforts attempted to build awareness of the importance of use of iodized salt as a health issue. Several other steps were taken by the government and partners to try to stabilize the iodine content in salt. First, there were attempts to improve packaging and storage practices—use of plastic lined bags for bulk packing of salt (50 kg) and storage in closed warehouses. Second, monitoring and reiodization at the five rail import sites were improved, with better warehousing, established labs, and closer monitoring. These efforts did improve household use of iodized salt, but iodine content remained somewhat inconsistent, as noted by variation in iodine content during household surveys.

### 3.4 Shifting consumer preference to refined salt

The program matured and was equipped with evidence that salt was iodized, but the consumer‐preferred phoda salt continued to be unlikely to meet standards. In response, the government focused on establishing ways to shift consumer preference to a more refined consumer packaged product that would stabilize the iodine content of salt. For many years, the coarser phoda salt continued to be the preferred salt, particularly in the mountains. Although STC was the sole importer of edible salt, there was such a high demand for phoda salt, especially in the mountains, and it was not possible to simply discontinue the supply. However, as more processed refined salt was introduced and available more widely, consumers began to increase their purchase of a more refined, cleaner packaged salt that they did not need to wash before using. The government and their partners, in particular UNICEF, developed an expanded communication campaign designed to reinforce an awareness of the importance of iodine to the health and development of children and to promote a “2‐child logo” refined packaged salt. The logo included a healthy child and was used in the communication campaign.

The new communication campaign, supported by UNICEF, consisted of several components. As early as 2000, the government worked with community‐based organizations (local nongovernmental organizations such as mothers' saving and credit groups, youth clubs, and others) to promote awareness of iodized salt using a social marketing model. This program was intensified in 2006 to focus on vulnerable areas. In 2004, there was a school‐based education program to increase awareness of iodine nutrition and use of iodized salt, along with a program for health workers that included adding iodine nutrition to maternal and child feeding program communications. Nationwide efforts also continued, celebrating the work done by the 52,000 female community health volunteers who promoted use of iodized salt. These efforts, starting in 1999, resulted in increased awareness of the value of the two‐child logo refined salt and an increase in its market share. (UNICEF, 2006) By changing consumer habits to purchase high quality packaged two‐child logo salt, use of coarser (less well iodized) salt became less common.

### 3.5 Demonstration of success: Trends in key indicators

**Figure 1:**
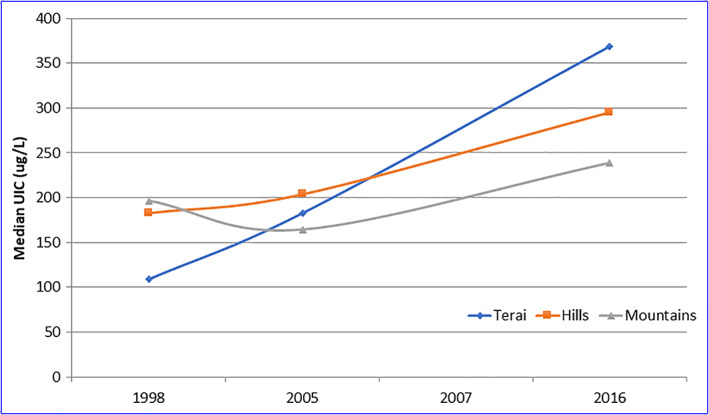
Trends in iodine status (school age children)—by ecological region

Comparing data from the four national micronutrient surveys conducted between 1998 and 2016, clear trends can be seen showing consistent progress towards the improvement in household use of iodized salt both at the national and subnational level, consistency of iodization, and in iodine intake. There are several striking aspects in reviewing these trends. First, there has been a positive trend in iodine status, as reflected by the median urinary iodine concentration (mUIC) of the population. Among school age children (SAC), the mUIC has increased from 144 to 314 ug/L between 1998 and 2016, with similar increases seen in all three ecological zones (Figure 1). It is noted that the national mUIC values and that in the Terai in 2016 were above 300 ug/L, which is the WHO/UNICEF/IGN cut‐off point, suggesting an excessive iodine intake (World Health Organization, 2007). Some subnational studies conducted in several districts of eastern Nepal during 2010–2012 have revealed mUIC in the range of 188–295 ug/L, indicating optimal iodine nutrition status (Table 1). In 1998, the iodine status of women of reproductive age (WRA) was 114 ug/L. In 2016, mUIC among WRA increased to 286 ug/L, which falls under the more than requirement category. In the Terai region, the mUIC among WRA (326 ug/L) indicates a risk of excessive iodine intake. The improvements in iodine status among SAC and WRA in 2016 are broadly distributed, and there are no subgroups at risk of suboptimal iodine intake. During 1994–1997, mUIC among pregnant women in one of the Terai districts (Sarlahi) was 96 ug/L, demonstrating iodine deficiency status (K J Schulze et al, 2003). A study conducted in three districts of eastern Terai districts in 2013–2014 found mUIC among pregnant women at 282 ug/L (Lalit Narayan Chaudhary et al, 2017). In 2016, the mUIC among pregnant women (where numbers were too small for analysis for many strata) was 241 ug/L; the only subgroup found to be below the WHO/UNICEF/IGN standard (mUIC between 150 and 249 ug/L to reflect optimal iodine intake) was in the far‐western region (mUIC = 134 ug/L). A study conducted in eastern Nepal found excessive iodine intake among infants (mUIC being 407 ug/L) but noted only around 7% subclinical hypothyroidism and less than 1 % overt hypothyroidism. The study suggested that thyroid in late infancy is already able to adapt high iodine intakes and, in most cases, maintain euthyroidism (Nepal Ashwini Kumar et al., 2015). The 2016 survey did not measure the iodine status of young children of 6–24 month for which the government has been using multiple micronutrient powders, which contain 90 ug of iodine. The program has covered half of the districts in Nepal, but the survey found only around 2% of children to have consumed multiple micronutrient powders at national level during the 7 days prior to the survey.

**Table 1:** Summary of trends in IDD indicators for Nepal

	NMNSS	NIDDSS	IDD survey	NMNSS
	1998 (%)	2005 (%)	2007 (%)	2016 (%)
**Household iodized salt**				
HHIS any iodine	83	95	100	97
HHIS adequate iodine (titration)	35	67	77	91
Percentage of HH using refined salt	10	33	53	88
Percentage of HH using phoda salt	63	42	41	16
Percentage of HH using 2‐child logo salt	na	38	53	89
Terai HHIS any iodine	79	na	na	97
Hill HHIS any iodine	87	na	na	95
Mnts HHIS any iodine	86	na	na	98
Percentage of subregions with percentage of HH using refined salt <50%	100	100	33	0
Terai percentage refined salt	8	na	51	96
Hills percentage refined salt	13	na	60	84
Mnts percentage refined salt	1	na	18	54
Terai percentage phoda (coarse) salt	45	na	37	5
Hills percentage phoda (coarse) salt	79	na	40	17
Mnts percentage phoda (coarse) salt	78	na	80	24
	NMNSS	NIDDSS	IDD survey	NMNSS
	1998	2005	2007	2016
**Population iodine status**				
Median urinary iodine concentration—mUIC ug/L (SAC)	144	188	203	314
Terai mUIC ug/L SAC	109	183	na	369
Hills mUIC ug/L SAC	183	204	na	295
Mountains mUIC ug/L SAC	197	165	na	239
Median urinary iodine concentration–mUIC ug/L (WRA)	114	na	na	286
Terai mUIC ug/L WRA	85	na	na	326
Hills mUIC ug/L WRA	143	na	na	241
Mountains mUIC ug/L WRA	169	na	na	280
Notes:				
NMNSS 1998 subregions = Terai, Hills, Mnts				
NIDDSS 2005 subregions = East and Central Terai, Hills, Mnts				
and all western Terai, hills, Mnts				

Abbreviations: IDD, iodine deficiency disorder; mUIC, median urinary iodine concentration; WRA, women of reproductive age.

**Figure 2:**
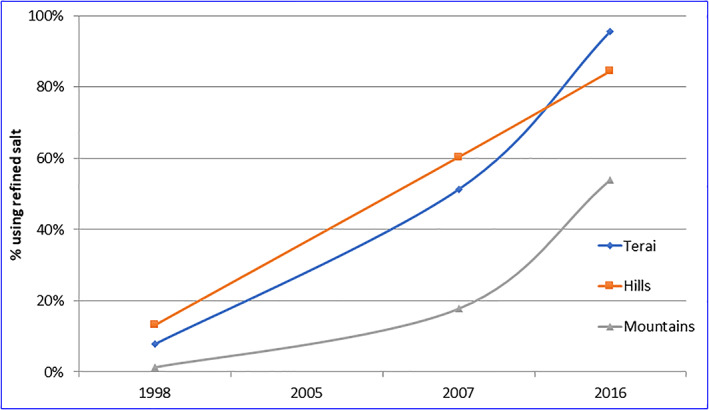
Trends in use of refined salt—by eco region

**Figure 3:**
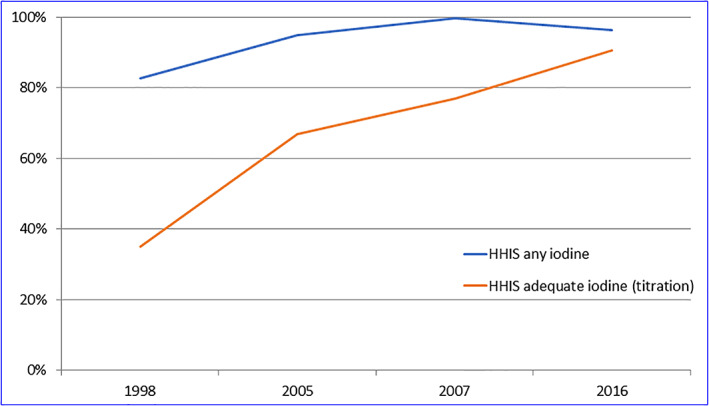
Trend in household iodized salt coverage

Second, there has been a marked shift from use of coarse (phoda) salt to use of a packaged refined salt (Figure 2). Overall, the proportion of households using refined salt was only 10% in 1998 and has increased to 88% in 2016, although the demand for refined salt in the mountains is still only about 56%. Third, in conjunction with the shift in consumer preference for salt type, there has been a clear increase in the quality of iodization as indicated by the iodine content of salt (Figure 3). In 2007, 77% of salt (in a subsample of 16% of households) contained >15 mg/kg, and this proportion increased to 91% by 2016. The 1998 and 2005 surveys analysed a 10% subsample of household salt by titration, with 35% >15 ppm in 1998 and 67% in 2005.

In 2016, these changes have not only taken place at the national, level, but are seen throughout the country among a number of subgroups. For example, among the 10 ethnic groups sampled, only one had a coverage below 80% use of refined salt (Hill Dalit: 74%). Over 80% of all salt samples were found to contain >15 mg/kg of iodine across all geographic regions, among urban/rural populations and across all ethnic groups, with only the lowest wealth quintile showing a lower percentage (78% for the lowest quintile).

There was a clear association between the iodine content and the type of salt used. Among refined salt samples, 97% contained >15 mg/kg, whereas the percentage of phoda salt with this level of iodine was only 46%. These data represent a dramatic progress for the salt iodization program, which has clearly resulted from a shift in consumer preference from coarse, phoda salt with inconsistent iodization, to the use of refined salt that is more consistently adequately iodized.

**Figure 4:**
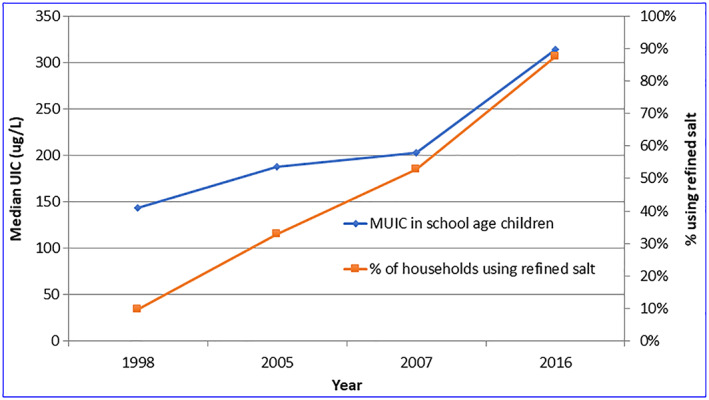
Association between iodine status (school age children) and proportion of households using refined salt. MUIC, median urinary iodine concentration

Refined salt has much greater retention of iodine than large crystal salt, and this feature has translated to a greater effectiveness of the salt iodization program (Figure 4).

**Figure 5:**
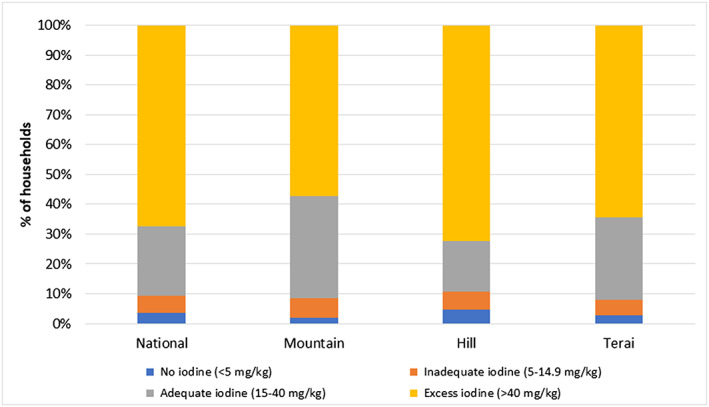
Iodine content in HHIS—by ecological region

Although the coverage of salt with adequate iodine content (>15 mg/kg) has increased, there is some reason for caution. In fact, the proportion of household iodized salt with excessive iodine content (>40 mg/kg) was 67.5% in 2016 (Figure 5), whereas only 23.2% of salt samples analysed were found to be within the optimal range (15–40 mg/kg). It is of note that the distribution of iodine in HHIS was similar in all ecological zones, with the highest percentage of excessive iodine in HHIS of the hills (72.4%), followed by the mountains (64.3%).

**Figure 6:**
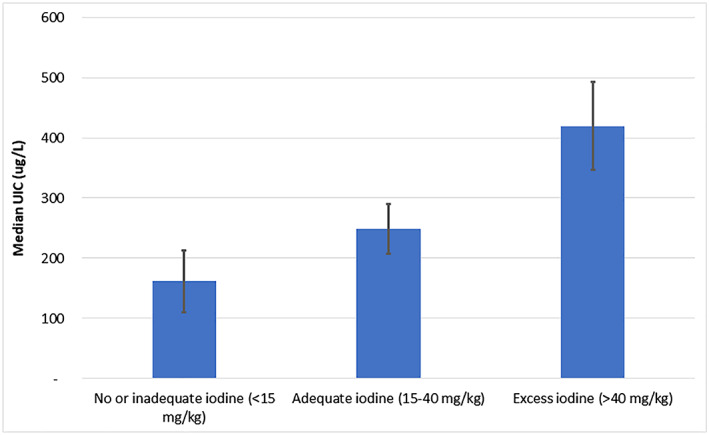
Population iodine status—by iodine content in salt

In 2016, there was a clear association between the iodine content in HHIS and the iodine status of the population (Figure 6). The mUIC value in SAC from households with low iodine in HHIS salt (<15 mg/kg) was 162 ug/L and increased to 249 ug/L among SAC from households whose HHIS content was adequate (15–40 mg/kg). Finally, among those SAC whose HHIS content was high (>40 mg/kg), the mUIC was 419 ug/L. These patterns were seen in earlier surveys, but the most recent data lead to several observations. First, even among households with low iodine in HHIS, the iodine status was classified as optimal suggesting that there may be other sources of iodine in the diet than solely the iodine from household iodized salt. Second, the iodine status of SAC in households with high iodine content led to an excessive iodine intake, and although this is likely not to translate to significant pathology of adverse consequences, it does inform potential program adjustments, for example, decreasing the standard for the iodine content in refined iodized salt.

## 4 DISCUSSION

The 2016 national micronutrient survey serves as a benchmark for progress to date, based on a number of program performance metrics. Clearly, efforts to increase the availability of iodized salt and improve iodine status have been successful. These achievements in the USI program are a direct reflection of the fact that the enabling environment has worked to improve consistency in iodization and use of iodized salt.

Nepal has in essence achieved optimal iodine nutrition, and its success should be celebrated. At the same time, it is important to understand key program elements that have led to the success and that can be strengthened in order to assure its sustainability. Many things likely contributed to this success:

Factors contributing to success

- *Political commitment and enabling environment*: There is historic evidence of a strong response by the government of Nepal to eliminate IDD. This is reflected in implementation of mandatory salt iodization by utilizing the power vested by different legislations, such as the 1957 Export and Import Control Act, the 1961 Essential Commodities Control Act, and the Competition Promotion, and Market Protection Act, 2063 (2007). Similarly, the government has maintained elimination of IDD as a component of the recent multisectoral nutrition strategy, and there is an ongoing structure to oversee the overall IDD elimination effort. Likewise, the government has been distributing packaged iodized salt at subsidized prices in remote mountains districts where consumption of adequately iodized salt by the most vulnerable population groups would otherwise be a major challenge.
- *Program Design and Implementation capacity*: The government established the STC as a sole agency for ensuring a supply of iodized salt and worked closely with other partners to provide technical insight for different program elements. A series of national USI plans of action enabled a systematic approach to the program with a clear modality of program oversight and well‐defined roles and responsibilities of different partners that strengthened overall coordination. Early trade negotiations with India helped stabilize the importation of iodized salt and helped establish the structure for storage, monitoring, and distribution. The government has been very effective in working closely with all stakeholders including donor partners, as evidenced by the collaboration with Japan International Cooperation Agency for warehouse development and with UNICEF and Nutrition International (formally Micronutrient Initiative) for communication and monitoring.
- *Monitoring and surveillance*: The government put in place monitoring structures for monitoring salt quality at different levels including at five major sites for importation. Indicators to assess the performance of the salt iodization program have been included in national surveys providing critical information on progress since 1998, providing good trend data on key indicators showing the success of the USI effort.
- *Social mobilization*: From the onset, there has been a successful effort to build awareness of the importance of iodized salt to health. There is a well‐documented communications approach that successfully shifted consumer preference to more refined salt that is more consistently iodized. This included use of community‐based organizations for social marketing and use of Women's Credit and Savings groups to assist distribution logistics. The shift in demand from phoda to refined salt had a direct impact on improving the supply of iodine and, in turn, on the iodine status of the population. The strategic focus of the communication on the “type” of salt that occurred along changes in consumer behaviour, for example, greater desire for high quality packaging, stands as a rare example of successful USI communications efforts.

In terms of the iodine status in the population and the risk of iodine deficiency, the picture is also clear. Overall, the iodine intake of the population has increased and likely has prevented deficiency, although there is a risk of excessive iodine in the diet.

## 5 FUTURE CHALLENGES AND STRATEGIC OPPORTUNITIES

### 5.1 Excessive iodine intake

WHO/UNICEF/IGN recommends that the iodine status of populations be based on the mUIC. It has been further designated that an optimal iodine intake is where a mUIC in SAC is in the range of 100–299 ug/L and excessive if the mUIC >300 ug/L (UNICEF, 2015). These levels are not as well defined for women of reproductive age, but for pregnant women, the optimal range is 150–249 ug/L, whereas the iodine intake is considered to be above requirements when the mUIC is between 250 and 499 ug/L and excessive >500 ug/L (WHO, 2013). With these criteria in mind, the 2016 survey shows that at the national level, SAC are currently classified in the “excessive” range, whereas pregnant women and women of child bearing age would be classified as “adequate” and “more than adequate,” respectively. Looking at the stratified results for several subgroups in the population, among the 34 strata listed, 19 (56%) were classified as excessive for SAC. Iodine intake was high in the central and western regions, in the Terai, and among a number of ethnic groups including the poorer Terai Janajati. For pregnant women, there were no strata with mUIC >500, but again, the sample size was small rendering these estimates imprecise. In addition, the assessment of iodine content of salt showed that mean iodine content for all salt samples was 44.1 ppm, and that 67.5% were >40 ppm—well above the expected level at the retail (30 ppm) and household (15 ppm) levels. The standards are based on an average per capita consumption of 10‐g salt per day, so an iodine content this high will provide more than three times the daily requirement.

These findings suggest that the current standard, combined with marked increase in use of a refined packaged product, may require an adjustment of the standard. The Nepal regulation/standard for iodized salt was developed when salt quality was poor, when packaging was suboptimal, and when losses of iodine were anticipated during transportation and storage. The shift toward a refined consumer packaged product has likely limited losses, allowing the accepted production level (50 ppm) to persist to the household level.

The STC continues to be the sole salt trade agency responsible for importing and distributing iodized salt. Salt imported by parties other than STC through the porous international boarder is regarded as unregulated and illegal by law. This in theory helps limit the amount of poorly iodized salt entering the country.

The current Nepal standard differs from the standard in India. This has resulted in most imported salt coming from larger sophistocated producers in India that meet the Nepal standard. Harmonizing the standard may result in a larger pool of producers supply Nepal's needs, potentially affecting the quality of iodization and increasing the amount of poorly iodized salt coming across the porous border.

### 5.2 Dietary changes

Currently, the evidence from the 2016 survey suggests a good correlation between the iodine levels of salt consumed in households and iodine status. However, dietary patterns change, and with a movement toward increased consumption of processed foods (including packaged soups), the situation in Nepal will need to be monitored closely. It may be useful, for example, to consider an assessment of processed food consumption and, more specifically, the contribution of these foods to salt consumption. Because salt for human consumption is primarily provided through STC, it is likely that virtually all processed foods produced in Nepal use iodized salt. However, shifting patterns of household salt use and introduction of new products may make it important to understand this contribution.

The disease patterns in Nepal have also changed, with a growing problem with chronic diseases including diabetes, heart disease, and hypertension. (Mohan & Prabhakaran, 2013) With the global movement toward salt reduction, the multisector action plan for the prevention and control of non‐communicable diseases (2014‐2020) of the government of Nepal has target of 30% relative reduction in mean population intake of salt (Government of Nepal, WHO, 2014–2020). Whereas it is relatively easy to adjust the standards for iodized salt to accommodate these changes, it is considerably more difficult to understand how effective a salt reduction recommendation is in reducing salt intake. This will require careful review and monitoring of salt reduction strategy implementation in the country.

### 5.3 Sustaining the enabling environment

The government of Nepal has been attentive to the issue of iodine deficiency for several decades and has included iodine program elements in the recent multisectoral nutrition strategy. In some countries, once certain benchmarks were reached, there was less interest in IDD, and in some instances, there was backsliding. Seemingly innocuous changes, for example, in the wording of legislation, can result in weakening of the IDD program (Codling et al, 2017). Other priorities can limit funding for critical elements of the program. None of this has happened in Nepal, and the current survey again suggests that the micronutrient component of the nutrition of women and children remains a high priority despite gains made. This suggests that the basic foundation for a sustained enabling environment is well established. With a shift in focus to a broader approach to nutrition, and on reduction in stunting, it will be critical to ensure that the monitoring of micronutrient deficiency continues so the changes noted above do not adversely affect the sustained elimination of IDD.

## 6 CONCLUSION

Nepal has effectively eliminated IDD as a public health problem through a successful USI program. The most recent survey confirms this success but suggests some consideration should be given to several program areas:

- Review and consider reduction in the standard for production and retail salt iodine levels. This should be done in consultation with all relevant stakeholders to ensure that imported salt meets the new standard, and that inadequately iodized salt coming across the porous border does not increase.
- Ensure the adequacy of ongoing monitoring of imported salt to track any changes in sources and quality. Complement this with periodic assessments of iodine status
- Consider a brief review of salt used in the processed food industry and the consumption of processed foods and condiments with a high salt content in Nepal—to anticipate the likely increasing trend and its implication for the USI program
- Establish a review mechanism that triangulates data on salt consumption, iodine status, and sources of salt in the diet, which will allow for rapid change in the iodine standard for salt in the event of successful salt reduction strategies

## CONFLICTS OF INTEREST

Naveen Paudyal, Sanjay Rijal, and Stanley Chitekwe, in their capacity working for UNICEF, support the government of Nepal's effort toward universal salt iodization but have no conflict of interest in developing this summary. The opinions and statements in this article are those of the authors and may not reflect official UNICEF policies. Kedar Parajuli, in his capacity working for government of Nepal to prevent and control iodine deficiency in Nepal, has no conflict of interest in developing this summary. Robin Houston, Chandra Pandav, Macha Raja Maharjan, and Jonathan Gorstein have all both undertaken consultancies with various agencies working in Nepal on nutrition issues, including in support of the USI program, but have no conflict of interest related to this paper.

## CONTRIBUTIONS

NP and SC contributed to the article through provision of reference material, editorial support, and drafting of some policy sections of the main text. JG, CP, and MRM contributed to the main text, data review, and editing. RH contributed by drafting the initial text, editing, and data presentation. KP provided input on the paper from the government IDD control program perspective.
